# MRI and PET findings with long‐term gantenerumab treatment: results from the DIAN‐TU trial

**DOI:** 10.1002/alz.094832

**Published:** 2025-01-09

**Authors:** Tammie L.S. Benzinger, Brian A. Gordon, Austin A. McCullough, Russ C. Hornbeck, Shaney Flores, Robert A. Koeppe, Clifford R. Jack, Yan Li, Eric McDade, Jorge J. Llibre‐Guerra, David B. Clifford, Susan Mills, Anna Santacruz, Guoqiao Wang, Charlene Supnet‐Bell, Laura Ibanez, Gregory Klein, Monika Baudler, Rachelle S. Doody, Geoffrey A. Kerchner, Alireza Atri, Randall J. Bateman, The DIAN‐TU Study Team

**Affiliations:** ^1^ Washington University in St. Louis, St. Louis, MO USA; ^2^ Knight Alzheimer Disease Research Center, St. Louis, MO USA; ^3^ Washington University in St. Louis, School of Medicine, St. Louis, MO USA; ^4^ Washington University School of Medicine, St. Louis, MO USA; ^5^ University of Michigan, Ann Arbor, MI USA; ^6^ Mayo Clinic, Rochester, MN USA; ^7^ Knight Alzheimer Disease Research Center, St Louis, MO USA; ^8^ Washington University in St. Louis School of Medicine, St. Louis, MO USA; ^9^ Washington University School of Medicine in St. Louis, St. Louis, MO USA; ^10^ F. Hoffmann‐La Roche Ltd, Basel Switzerland; ^11^ Banner Alzheimer’s Institute, Phoenix, AZ USA

## Abstract

**Background:**

Anti‐amyloid monoclonal antibody therapies for AD have documented plaque removal on amyloid PET scans. We evaluate the findings for amyloid PET, tau PET, FDG PET and volumetric MRI over the course the open label extension (OLE) for the DIAN‐TU gantenerumab treatment, compared to the last imaging obtained prior to the OLE (to evaluate for potential rebound effects) and in comparison to longitudinal imaging in the DIAN Observational study.

**Method:**

This double‐blind, phase 2/3 trial (2012‐2019), followed by open‐label extension (OLE), investigated varying gantenerumab doses up to 1500 mg SQ q2 weeks [NCT NCT01760005]. We report long‐term amyloid‐plaque removal by PET and findings for metabolic FDG PET and tau PET, and additionally changes of atrophy on volumetric MRI. Imaging measures were secondary outcome analyses. Comparison is made between the treatment cohort and controls (DB placebo participants who didn’t enter OLE, plus external controls from the DIAN OBS). Estimated years to onset was included as a covariate. The gap period of ∼1 year is specifically analyzed to estimate any “rebound” effect on amyloid or tau accumulation or neurodegeneration. Volumetric MRI measures include hippocampal volumes, lateral ventricular volumes, and white matter hyperintensities.

**Result:**

In participants who were asymptomatic at baseline and treated with gantenerumab (n = 53), amyloid‐plaque removal measured by PIB‐PET in Centiloids was dose‐dependent. Few participants had reached completely negative levels (below 24). No statistical significance was found for FDG PET, tau PET, or volumetric MRI, including hippocampal volumes. Impact of the gap period of ∼1 year (gap in treatment) on the rate of amyloid accumulation compared to the rate of accumulation predicted in observational studies of this cohort is under evaluation, as are the other imaging measures.

**Conclusion:**

While no overall effect was observed in the total group for non‐amyloid measures, results suggest dose dependent clearance of amyloid plaque in asymptomatic DIAD mutation carriers following long‐term, high‐dose gantenerumab. The potential “rebound effect” of elevated amyloid accumulation for participants for whom there was a significant gap in therapy between the trial and the OLE is under evaluation and will be presented, as will the variables for FDG PET, tau PET, and volumetric MRI.

